# 2,4-Di­bromo-6-{[(5-chloro-2-methyl­phen­yl)imino]­meth­yl}phenol

**DOI:** 10.1107/S1600536813017558

**Published:** 2013-06-29

**Authors:** Yunfa Zheng

**Affiliations:** a Department of Chemistry, Lishui University, Lishui 323000, People’s Republic of China

## Abstract

In the mol­ecular structure of the title Schiff base, C_14_H_10_Br_2_ClNO, the chloro­phenyl ring and di­bromo­phenol ring are almost coplanar; the dihedral angle between the planes of the two rings is 10.50 (18)°. There is an intra­molecular O—H⋯N hydrogen bond, with an O⋯N distance of 2.576 (4)Å. The crystal structure is stabilized by π–π stacking of neighbouring aromatic rings along the *b*-axis direction [centroid–centroid distance = 3.6896 (5) Å].

## Related literature

For general background, see: Siddiqui *et al.* (2006 ▶); Fukuda *et al.* (2009 ▶); Elmali & Elerman (1998 ▶); Karakas *et al.* (2004 ▶); Ebrahimipour *et al.* (2012 ▶). For the similar Schiff base structures, see: Zhou *et al.* (2009 ▶); Atalay *et al.* (2008 ▶).
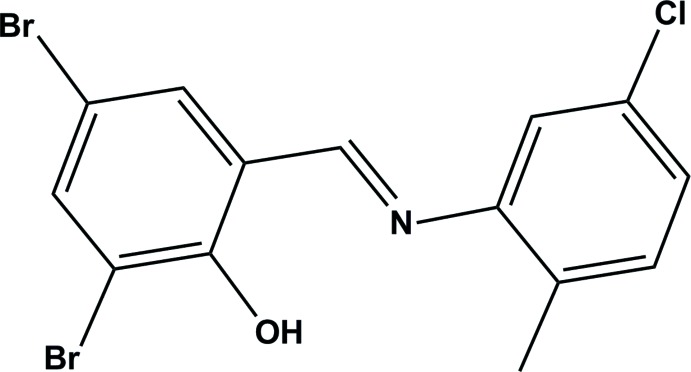


## Experimental

### 

#### Crystal data

C_14_H_10_Br_2_ClNO
*M* *_r_* = 403.48
Monoclinic, 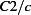

*a* = 31.603 (5) Å
*b* = 6.1828 (10) Å
*c* = 14.890 (2) Å
β = 102.594 (15)°
*V* = 2839.4 (8) Å^3^
*Z* = 8
Mo *K*α radiation
μ = 5.89 mm^−1^
*T* = 295 K
0.38 × 0.35 × 0.30 mm

#### Data collection

Bruker APEXII CCD diffractometer
Absorption correction: multi-scan (*SADABS*; Sheldrick, 2003 ▶) *T*_min_ = 0.123, *T*_max_ = 0.171
5369 measured reflections
2600 independent reflections
1839 reflections with *I* > 2σ(*I*)
*R*_int_ = 0.035

#### Refinement

*R*[*F* ^2^ > 2σ(*F* ^2^)] = 0.038
*wR*(*F* ^2^) = 0.082
*S* = 1.00
2600 reflections
174 parameters
H-atom parameters constrained
Δρ_max_ = 0.41 e Å^−3^
Δρ_min_ = −0.37 e Å^−3^

### 

Data collection: *SMART* (Bruker, 2004 ▶); cell refinement: *SAINT* (Bruker, 2004 ▶); data reduction: *SAINT*; program(s) used to solve structure: *SHELXS97* (Sheldrick, 2008 ▶); program(s) used to refine structure: *SHELXL97* (Sheldrick, 2008 ▶); molecular graphics: *ORTEP-3* (Farrugia, 2012 ▶); software used to prepare material for publication: *SHELXL97*.

## Supplementary Material

Crystal structure: contains datablock(s) I, global. DOI: 10.1107/S1600536813017558/rk2407sup1.cif

Structure factors: contains datablock(s) I. DOI: 10.1107/S1600536813017558/rk2407Isup2.hkl

Supplementary material file. DOI: 10.1107/S1600536813017558/rk2407Isup3.cml

Additional supplementary materials:  crystallographic information; 3D view; checkCIF report

## Figures and Tables

**Table 1:** Hydrogen-bond geometry (Å, °)

*D*—H⋯*A*	*D*—H	H⋯*A*	*D*⋯*A*	*D*—H⋯*A*
O1—H1⋯N1	0.82	1.85	2.576 (4)	147

**Fig. 1.**
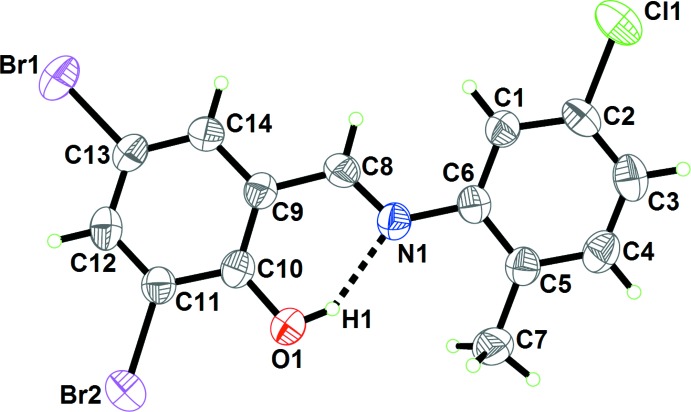
Molecular structure of the title compound with the atom numbering scheme. Displacement ellipsoids are drawn at 50% probability level. H atoms are presented as a small spheres of arbitrary radius. Dashed line indicates intramolecular hydrogen bond.

**Fig. 2.**
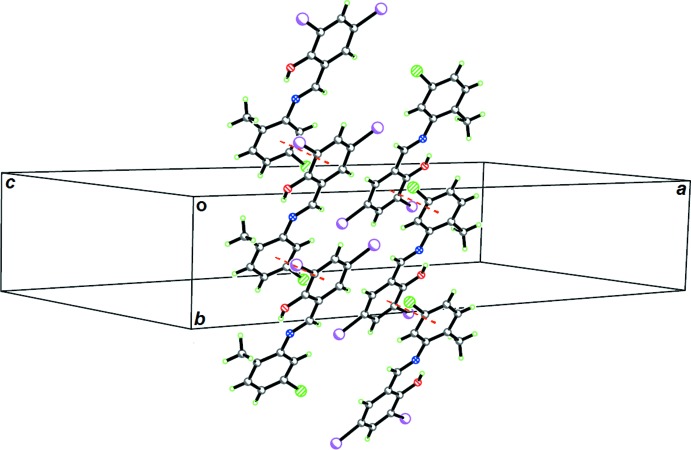
The π···π stacking of the title compound along the b axis.

**Fig. 3.**
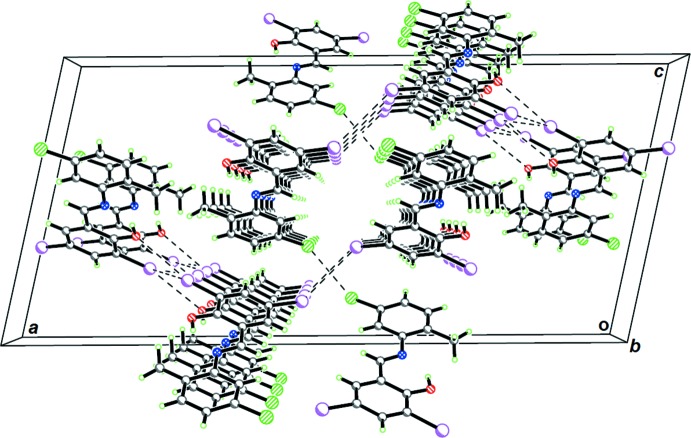
A packing diagram of the title compound, viewed along the b axis, showing the centrosymmetric arrangement.

## supplementary crystallographic information

### Comment

The Schiff bases derived from salicylaldehyde and methylaniline with various alkyl or halogen substituents have demonstrated potential application in pharmacal field, which have being tested for their antitumor, antimicrobial and antiviral activities (Siddiqui *et al.*, 2006). Schiff base compounds were also studied with respect to photochromic fluorescence materials (Fukuda *et al.*, 2009; Elmali *et al.*, 1998) and photochromic nonlinear optical materials (Karakas *et al.*, 2004). Moreover, Schiff bases have significant importance in the development of Schiff base metal complexes, because Schiff base ligands are potentially capable of forming stable complexes by coordination of metal ions with their oxygen and nitrogen donors (Ebrahimipour *et al.*, 2012). As an extension work on the structural characterization of Schiff base compounds, the title compound is reported.

The molecule of title compound adopts an *E* configuration, with a C6–N1═C8–C9 torsion angle of 178.4 (3)°. The bond distance of N1═C8 at 1.266 (4)Å is typical of a double bond, which is comparable to those found in similar structures (1.275 (4)Å, Zhou *et al.*, 2009; 1.264 (10)Å, Atalay *et al.*, 2008). The average bond lengths of C–Br at 1.890 (4)Å is longer than that of C–Cl at 1.744 (4)Å due to the radius of Br atom is bigger than that of Cl atom. It is noteworthy to note that H1 atom bonded to O1 is involved in O1–H1···N1 intramolecular hydrogen bond, which resulted in formation of six-membered ring (O1–H1···N1═C8–C9-C10) (Fig. 1). The dibromophenol ring is almost coplanar with the chlorophenyl ring with the dihedral angle between the two planes is 10.50 (18)°. Furthermore, the aromatic ring in the molecule is nearly parallel to the aromatic ring of its neighboring molecule with a ring-to-ring distance of 3.4715 (5)Å (3.6896 (5)Å) and an off-centre angle of 21.98°, indicating a weak π···π stacking interaction between the aromatic rings (Fig. 2). The packing diagram of the title compound shown stacks are arranged in a centrosymmetric manner and a *C*_2_ axis passing through the middle point of *ac* plane (Fig. 3).

### Experimental

A mixture of 5-chloro-2-methylaniline (1.42 g, 10 mmol), 3,5-dibromo-2-hydroxybenzaldehyde (2.80 g, 10 mmol) in 50 ml CH_2_Cl_2_ was refluxed under an Ar atmosphere for about 6 h to yield a yellow precipitate. The product was collected by filtration and washed with cold ethanol to give Schiff base compoud in 92.2% yield (3.55 g). The yellow single crystals suitable for X-ray analysis were grown from CH_2_Cl_2_ / absolute ethanol (3 / 2) systems by slow evaporation of the solvents at room temperature over a period of about one week.

### Refinement

Hydrogen atoms for the carbon atoms were placed in geometrically idealized positions and constrained to ride on their parent with C–H = 0.96Å and 0.93Å for methyl and aryl type H-atoms, respectively, and refined in a riding mode with *U*_iso_(H) = 1.2*U*_eq_(C) for aromatic H and *U*_iso_(H) = 1.5*U*_eq_(C) for methyl H.

### Crystal data

**Table:**

C_14_H_10_Br_2_ClNO	*F*(000) = 1568
*M**_r_* = 403.48	*D*_x_ = 1.888 Mg m^−^^3^
Monoclinic, *C*2/*c*	Mo *K*α radiation, λ = 0.71073 Å
Hall symbol: -C 2yc	Cell parameters from 1332 reflections
*a* = 31.603 (5) Å	θ = 3.2–29.4°
*b* = 6.1828 (10) Å	µ = 5.89 mm^−^^1^
*c* = 14.890 (2) Å	*T* = 295 K
β = 102.594 (15)°	Block, yellow
*V* = 2839.4 (8) Å^3^	0.38 × 0.35 × 0.30 mm
*Z* = 8	

### Data collection

**Table:**

Bruker APEXII CCD diffractometer	2600 independent reflections
Radiation source: fine-focus sealed tube	1839 reflections with *I* > 2σ(*I*)
Graphite monochromator	*R*_int_ = 0.035
φ– and ω–scans	θ_max_ = 25.4°, θ_min_ = 3.4°
Absorption correction: multi-scan (*SADABS*; Sheldrick, 2003)	*h* = −38→37
*T*_min_ = 0.123, *T*_max_ = 0.171	*k* = −7→7
5369 measured reflections	*l* = −12→17

### Refinement

**Table:**

Refinement on *F*^2^	Primary atom site location: structure-invariant direct methods
Least-squares matrix: full	Secondary atom site location: difference Fourier map
*R*[*F*^2^ > 2σ(*F*^2^)] = 0.038	Hydrogen site location: inferred from neighbouring sites
*wR*(*F*^2^) = 0.082	H-atom parameters constrained
*S* = 1.00	*w* = 1/[σ^2^(*F*_o_^2^) + (0.033*P*)^2^] where *P* = (*F*_o_^2^ + 2*F*_c_^2^)/3
2600 reflections	(Δ/σ)_max_ < 0.001
174 parameters	Δρ_max_ = 0.41 e Å^−^^3^
0 restraints	Δρ_min_ = −0.37 e Å^−^^3^

### Special details

**Table:**

Geometry. All s.u.'s (except the s.u. in the dihedral angle between two l.s. planes) are estimated using the full covariance matrix. The cell s.u.'s are taken into account individually in the estimation of s.u.'s in distances, angles and torsion angles; correlations between s.u.'s in cell parameters are only used when they are defined by crystal symmetry. An approximate (isotropic) treatment of cell s.u.'s is used for estimating s.u.'s involving l.s. planes.
Refinement. Refinement of *F*^2^ against ALL reflections. The weighted *R*-factor *wR* and goodness of fit *S* are based on *F*^2^, conventional *R*-factors *R* are based on *F*, with *F* set to zero for negative *F*^2^. The threshold expression of *F*^2^ > σ(*F*^2^) is used only for calculating *R*-factors(gt) *etc*. and is not relevant to the choice of reflections for refinement. *R*-factors based on *F*^2^ are statistically about twice as large as those based on *F*, and *R*-factors based on ALL data will be even larger.

### Fractional atomic coordinates and isotropic or equivalent isotropic displacement parameters (Å^2^)

**Table:**

	*x*	*y*	*z*	*U*_iso_*/*U*_eq_	
Br1	0.032809 (16)	0.04959 (8)	0.15977 (4)	0.07156 (19)	
Br2	0.214268 (14)	0.15664 (7)	0.23337 (3)	0.06296 (18)	
Cl1	0.04381 (4)	1.43342 (19)	−0.16015 (9)	0.0733 (4)	
O1	0.18961 (8)	0.5562 (4)	0.1191 (2)	0.0543 (7)	
H1	0.1827	0.6566	0.0829	0.081*	
N1	0.13813 (10)	0.8234 (4)	0.0161 (2)	0.0398 (7)	
C1	0.09405 (12)	1.1128 (6)	−0.0732 (3)	0.0425 (9)	
H1A	0.0687	1.0563	−0.0605	0.051*	
C2	0.09307 (13)	1.2987 (6)	−0.1244 (3)	0.0446 (10)	
C3	0.12950 (15)	1.3817 (7)	−0.1455 (3)	0.0525 (11)	
H3	0.1281	1.5071	−0.1805	0.063*	
C4	0.16871 (14)	1.2770 (6)	−0.1143 (3)	0.0511 (11)	
H4	0.1936	1.3326	−0.1293	0.061*	
C5	0.17164 (12)	1.0909 (6)	−0.0610 (3)	0.0417 (9)	
C6	0.13395 (12)	1.0104 (6)	−0.0405 (2)	0.0371 (9)	
C7	0.21455 (13)	0.9824 (7)	−0.0262 (3)	0.0567 (11)	
H7A	0.2227	0.9965	0.0395	0.085*	
H7B	0.2361	1.0493	−0.0535	0.085*	
H7C	0.2122	0.8319	−0.0424	0.085*	
C8	0.10618 (13)	0.7192 (6)	0.0326 (3)	0.0416 (9)	
H8	0.0782	0.7678	0.0079	0.050*	
C9	0.11218 (12)	0.5254 (5)	0.0891 (2)	0.0373 (9)	
C10	0.15431 (12)	0.4507 (6)	0.1287 (2)	0.0381 (9)	
C11	0.15814 (12)	0.2580 (6)	0.1790 (2)	0.0391 (9)	
C12	0.12263 (13)	0.1421 (6)	0.1892 (2)	0.0433 (10)	
H12	0.1260	0.0137	0.2225	0.052*	
C13	0.08149 (12)	0.2177 (6)	0.1493 (3)	0.0426 (9)	
C14	0.07621 (12)	0.4088 (6)	0.1011 (3)	0.0435 (10)	
H14	0.0485	0.4603	0.0764	0.052*	

### Atomic displacement parameters (Å^2^)

**Table:**

	*U*^11^	*U*^22^	*U*^33^	*U*^12^	*U*^13^	*U*^23^
Br1	0.0586 (3)	0.0699 (3)	0.0919 (4)	−0.0168 (2)	0.0287 (3)	0.0172 (3)
Br2	0.0527 (3)	0.0652 (3)	0.0679 (3)	0.0080 (2)	0.0066 (2)	0.0210 (2)
Cl1	0.0641 (8)	0.0718 (8)	0.0766 (9)	0.0211 (6)	−0.0010 (6)	0.0219 (6)
O1	0.0407 (16)	0.0495 (17)	0.070 (2)	−0.0082 (13)	0.0062 (14)	0.0174 (14)
N1	0.0429 (19)	0.0362 (17)	0.0400 (19)	−0.0028 (15)	0.0082 (15)	0.0012 (15)
C1	0.040 (2)	0.039 (2)	0.047 (2)	−0.0042 (18)	0.0064 (19)	0.0021 (19)
C2	0.048 (2)	0.043 (2)	0.039 (2)	0.0113 (19)	0.0019 (19)	0.0023 (19)
C3	0.070 (3)	0.043 (2)	0.043 (3)	0.001 (2)	0.010 (2)	0.0080 (19)
C4	0.055 (3)	0.055 (3)	0.046 (3)	−0.009 (2)	0.017 (2)	0.005 (2)
C5	0.047 (2)	0.041 (2)	0.037 (2)	−0.0023 (18)	0.0093 (19)	0.0002 (18)
C6	0.045 (2)	0.031 (2)	0.035 (2)	−0.0017 (17)	0.0076 (18)	−0.0045 (16)
C7	0.044 (2)	0.071 (3)	0.056 (3)	−0.002 (2)	0.013 (2)	0.013 (2)
C8	0.038 (2)	0.037 (2)	0.050 (2)	0.0060 (18)	0.0096 (19)	0.0013 (18)
C9	0.042 (2)	0.034 (2)	0.038 (2)	−0.0011 (17)	0.0124 (18)	−0.0015 (17)
C10	0.044 (2)	0.034 (2)	0.038 (2)	−0.0053 (18)	0.0106 (18)	−0.0038 (17)
C11	0.043 (2)	0.037 (2)	0.037 (2)	0.0001 (17)	0.0105 (18)	0.0001 (18)
C12	0.061 (3)	0.035 (2)	0.038 (2)	0.0003 (19)	0.019 (2)	0.0013 (17)
C13	0.042 (2)	0.045 (2)	0.042 (2)	−0.0095 (19)	0.0130 (19)	−0.0016 (19)
C14	0.040 (2)	0.043 (2)	0.050 (2)	0.0046 (18)	0.0132 (19)	0.0041 (19)

### Geometric parameters (Å, º)

**Table:**

Br1—C13	1.891 (4)	C5—C6	1.385 (5)
Br2—C11	1.889 (4)	C5—C7	1.500 (5)
Cl1—C2	1.744 (4)	C7—H7A	0.9600
O1—C10	1.327 (4)	C7—H7B	0.9600
O1—H1	0.8200	C7—H7C	0.9600
N1—C8	1.266 (4)	C8—C9	1.453 (5)
N1—C6	1.420 (4)	C8—H8	0.9300
C1—C2	1.376 (5)	C9—C14	1.390 (5)
C1—C6	1.400 (5)	C9—C10	1.411 (5)
C1—H1A	0.9300	C10—C11	1.398 (5)
C2—C3	1.358 (6)	C11—C12	1.367 (5)
C3—C4	1.385 (6)	C12—C13	1.387 (5)
C3—H3	0.9300	C12—H12	0.9300
C4—C5	1.389 (5)	C13—C14	1.373 (5)
C4—H4	0.9300	C14—H14	0.9300
			
C10—O1—H1	109.5	H7A—C7—H7C	109.5
C8—N1—C6	123.7 (3)	H7B—C7—H7C	109.5
C2—C1—C6	118.7 (4)	N1—C8—C9	121.5 (3)
C2—C1—H1A	120.7	N1—C8—H8	119.2
C6—C1—H1A	120.7	C9—C8—H8	119.2
C3—C2—C1	121.8 (4)	C14—C9—C10	120.0 (3)
C3—C2—Cl1	119.6 (3)	C14—C9—C8	119.7 (3)
C1—C2—Cl1	118.6 (3)	C10—C9—C8	120.3 (3)
C2—C3—C4	119.1 (4)	O1—C10—C11	120.0 (3)
C2—C3—H3	120.4	O1—C10—C9	122.2 (3)
C4—C3—H3	120.4	C11—C10—C9	117.8 (3)
C3—C4—C5	121.5 (4)	C12—C11—C10	121.9 (3)
C3—C4—H4	119.3	C12—C11—Br2	119.7 (3)
C5—C4—H4	119.3	C10—C11—Br2	118.4 (3)
C6—C5—C4	118.0 (3)	C11—C12—C13	119.4 (3)
C6—C5—C7	121.3 (3)	C11—C12—H12	120.3
C4—C5—C7	120.7 (4)	C13—C12—H12	120.3
C5—C6—C1	120.9 (3)	C14—C13—C12	120.6 (3)
C5—C6—N1	116.8 (3)	C14—C13—Br1	120.5 (3)
C1—C6—N1	122.3 (3)	C12—C13—Br1	118.9 (3)
C5—C7—H7A	109.5	C13—C14—C9	120.3 (3)
C5—C7—H7B	109.5	C13—C14—H14	119.9
H7A—C7—H7B	109.5	C9—C14—H14	119.9
C5—C7—H7C	109.5		
			
C9—C8—N1—C6	178.4 (3)		

### Hydrogen-bond geometry (Å, º)

**Table:**

*D*—H···*A*	*D*—H	H···*A*	*D*···*A*	*D*—H···*A*
O1—H1···N1	0.82	1.85	2.576 (4)	147
